# Pushing the limits of LDL cholesterol: Emerging paradigms in cardiovascular risk reduction

**DOI:** 10.1016/j.tcm.2025.07.002

**Published:** 2025-07-13

**Authors:** Dadmehr Yaghoubi, Tamer Sallam

**Affiliations:** a Division of Cardiology, Department of Medicine, David Geffen School of Medicine, University of California, Los Angeles, CA, USA; b Center for preventive Cardiology & Cardiometabolic Health, University of California, Los Angeles, CA, USA; c Department of Physiology, University of California, Los Angeles, CA, USA; d Molecular Biology Institute, University of California, Los Angeles, CA, USA

**Keywords:** Cholesterol, Coronary artery disease, Statins, LDL

## Abstract

LDL cholesterol (LDL-C) has long been recognized as a primary contributor to cardiovascular disease. Over time, guideline-recommended LDL-C targets have varied considerably, trending toward progressively more aggressive therapeutic goals. The focus on residual risk reduction and development of novel therapies—including PCSK9 inhibitors, siRNA-based treatments, and ANGPTL3 inhibitors—have significantly expanded the options for achieving unprecedentedly low LDL-C levels. Given the fundamental role of cholesterol in cellular function, it has been long been postulated that very low cholesterol could be associated with adverse events. In this review, we dissect the benefits and potential risks of very low LDL-C levels. We begin by providing an overview of the evolution of LDL-C targets in previous guidelines and highlighting therapies—including newer agents—used for aggressive LDL-C lowering. Drawing on clinical and genetic evidence, we then examine the benefits and risks associated with achieving very low LDL-C levels. Finally, we present a practical framework for balancing the potential risks and benefits of intensive LDL-C reduction in patient care.

## Introduction

Cholesterol forms the epicenter of major forms of atherosclerotic cardiovascular disease. For this reason, cholesterol management has been a central issue in cardiovascular health, with low-density lipoprotein cholesterol (LDL-C) emerging as the primary treatment target [1]. Despite consensus on its role in cardiovascular disease, optimal LDL-C levels and potential risks of aggressive reduction remain points of debate. The question of ‘*how low should LDL-C be?*’ has gained increasing relevance due to the development of potent LDL-C lowering therapies and interest in addressing residual cardiovascular risk.

Over the past century, discussions surrounding the role of cholesterol have been both prominent and, at times, controversial. Cholesterol has been long recognized as a key component in several physiological processes, including steroid hormone production [2], bile acid synthesis [3], maintaining cell membrane integrity [4], and modulating immune function [5,6]. Given its essential role in cellular homeostasis, concerns arose that lowering cholesterol might adversely impact health, leaving individuals susceptible to hormone imbalances, nutrient deficiencies, infections, and malignancies. In fact, even half a century following Nikolay Anichkov’s demonstration in 1913 that feeding rabbits a cholesterol-rich diet increased their risk of heart disease, there remained substantial opposition to the idea that cholesterol is linked to heart disease or that lowering cholesterol provides cardioprotection [7]. This viewpoint began to shift after the Framingham Heart Study, which identified a strong association between elevated cholesterol and an increased risk of cardiovascular disease [8]. Elucidating the molecular basis of LDL receptor regulation by Brown and Goldstein was a groundbreaking discovery that lent strong support to the cholesterol hypothesis [9,10] and earned them the 1985 Nobel Prize. Later, the Scandinavian Simvastatin Survival Study (4S) provided direct evidence that lowering LDL-C cholesterol through treatment with simvastatin significantly reduces the risk of cardiovascular events and mortality [11]. As a result, the skepticism about aggressive LDL-C reduction diminished. While most clinical trials did not intentionally treat patients to specific LDL-C targets, guidelines have repeatedly emphasized LDL-C target goals that have drifted downwards over time. At times, clinical guidelines have approached very low LDL-C with caution. For example, the 2013 ACC/AHA cholesterol guidelines suggested decreasing statin dosing when 2 consecutive values of LDL-C are <40 mg/Dl [12]. This recommendation was not based on outcomes data or adverse events for LDL-C below that level, rather based on the approach taken in a number of randomized controlled trials [12]. Today, with clear benefits in reducing coronary artery disease, clinicians are exploring more aggressive LDL-C targets, sometimes as low as 30 mg/dL [13], raising the question of *how low is too low* and whether the potential benefits outweigh the risks for all individuals. In this review, we disentangle key considerations for management of very low LDL-C. We will first provide an overview of the evolution of LDL-C targets in prior guidelines and highlight therapies—including newer agents—that can be used for aggressive LDL-C lowering. Integrating clinical and genetic evidence, we will discuss benefits and risks of very low LDL-C reduction. Finally, we provide a practical framework for balancing potential risks and benefits of LDL lowering.

## Evolution of LDL-C threshold in guidelines

**Fig. 1.**
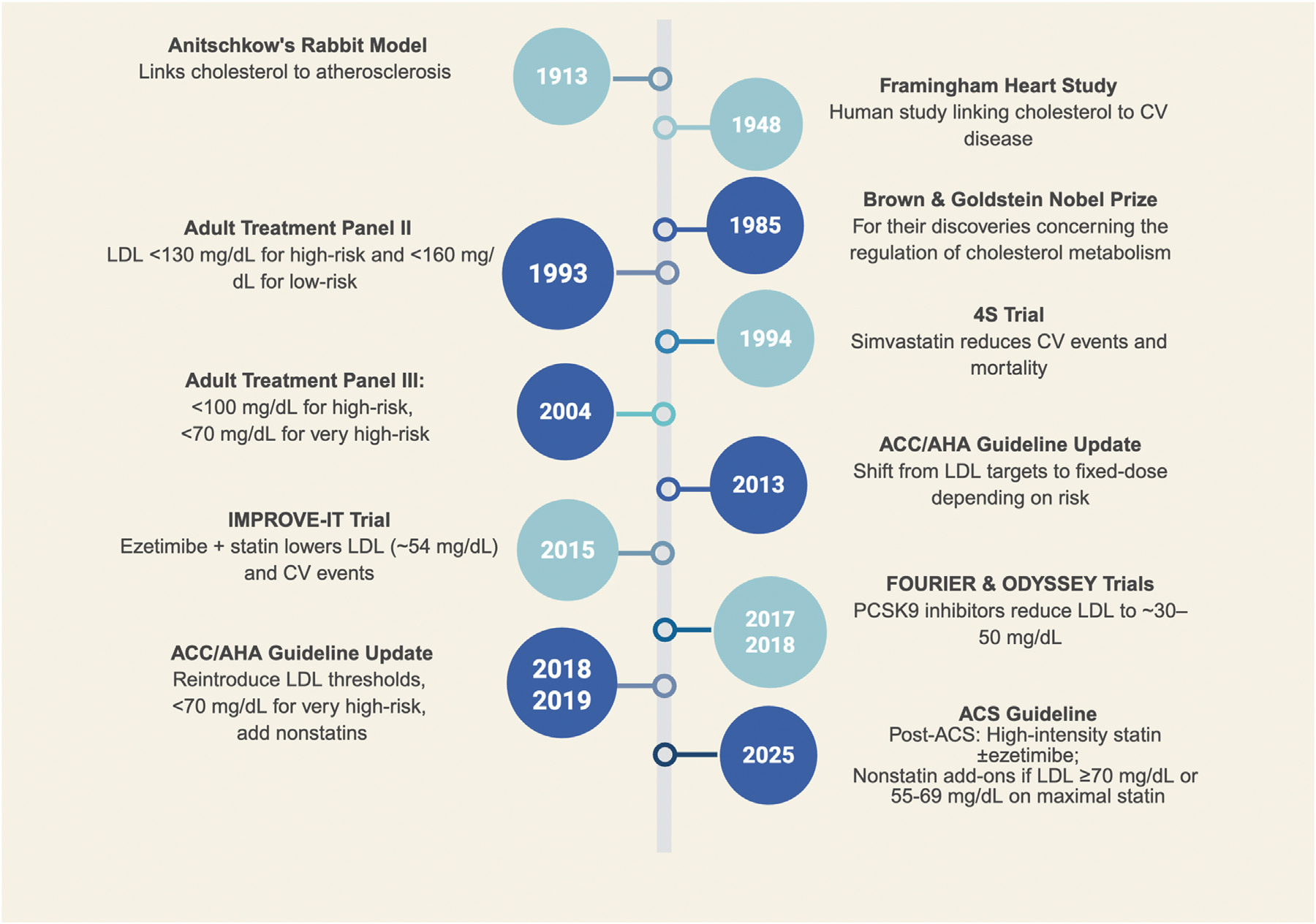
The timeline of key events in LDL Cholesterol management. CV=Cardiovascular

There are no clear paradigms in the literature that define what constitutes ‘low’ or ‘very low’ cholesterol. It’s thought that LDL-*C* < 50–70mg/dl represents the 5–10th percentile [14,15]. Some studies referred to LDL-C <40 mg/dl as ‘low’ or ‘very low’. Others used threshold of <20 or 25 mg/dl to define ‘very low’ or ‘extremely low’ LDL-C. Thus, it is important to keep in mind that these definitions are somewhat arbitrary. A key consideration, however, is that the cutoff for LDL-C thresholds have evolved significantly over decades (Fig. 1).

In 1993, the National Cholesterol Education Program (NCEP) Adult Treatment Panel (ATP) II recognized LDL-C as the primary target for cholesterol-lowering therapy, setting <130 mg/dL for high-risk patients (those with established atherosclerotic cardiovascular disease) and <160 mg/dL for low-risk individuals (fewer than two risk factors). This laid the groundwork for ATP III which in 2004 recommended LDL-*C* < 100 mg/dL for high-risk patients, with an optional <70 mg/dL for very high-risk individuals—underscoring emerging evidence that lower LDL-C was associated with fewer cardiovascular events [16].

The 2013 ACC/AHA Cholesterol guidelines shifted the focus from strict LDL-C targets to fixed-intensity statin therapy based on overall atherosclerotic cardiovascular disease (ASCVD) risk, an approach informed by randomized controlled trials showing that the intensity of statin therapy rather than a specific LDL-C “number” drove reductions in cardiovascular events [17]. By the 2018 ACC/AHA guidelines for primary prevention, statin therapy was recommended for young adults (20–39 years) if LDL-*C* ≥ 160 mg/dL and additional risk factors, while for adults aged 40–75 years without diabetes, decisions were guided by a 10-year ASCVD risk calculation [18].

For secondary prevention, the emphasis is on reducing LDL-C below 70 mg/dL in patients with established atherosclerotic cardiovascular disease (ASCVD). The 2022 ACC expert consensus statement goes further in its recommendations for very high-risk individuals, suggesting additional lipid-lowering measures if LDL-C remains ≥55 mg/dL on maximal statin therapy. In the context of acute coronary syndrome (ACS), the 2025 guideline updates recommend high-intensity statin therapy for all patients, with the option to add ezetimibe from the outset. Additionally, for patients whose LDL-C is ≥70 mg/dL on maximally tolerated statins, a non-statin agent is advised. For very high-risk patients with LDL-C between 55 and 70 mg/dL despite maximal statin therapy, further intensification of therapy may be warranted [19,20].

The 2019 ESC/EAS guidelines recommend an LDL-C goal of <55 mg/dL for very high-risk patients defined as patients with documented ASCVD, severe CKD (eGFR <30), diabetes accompanied by organ damage or multiple risk factors, high 10-year SCORE risk (≥10 %), or familial hypercholesterolemia with additional major risk factors. The guidelines go on to suggest aiming for an LDL-C of <40 mg/dL if a second vascular event occurs within two years, reinforcing the principle that lowering LDL-C to increasingly stringent thresholds reduces cardiovascular events, especially in high-risk and very high-risk groups [21].

Aligned with the major cardiovascular societies, the American Diabetes Association recommends statin therapy for most adults with diabetes—using moderate–intensity statins for primary prevention (ages 40–75) and high–intensity therapy to reach LDL–*C* < 70 mg/dL in those at higher risk or with ASCVD, adding non-statins if targets are not met [22]. The American Association of Clinical Endocrinology stratifies diabetic ASCVD risk into high (<100 mg/dL), very high (<70 mg/dL), and extreme (<55 mg/dL) LDL-C goals [23]. The National Lipid Association also endorses LDL–C targets of <70 mg/dL for very high-risk and <55 mg/dL for extreme–risk patients based on the 2022 ACC expert consensus [19]. All three underscore regular lipid monitoring to ensure adherence and achievement of these goals. Thus, in summary both national and international society guidelines have evolved to advocating to lower LDL cholesterol.

## Pharmacotherapies for achieving very low LDL-C

**Fig. 2.**
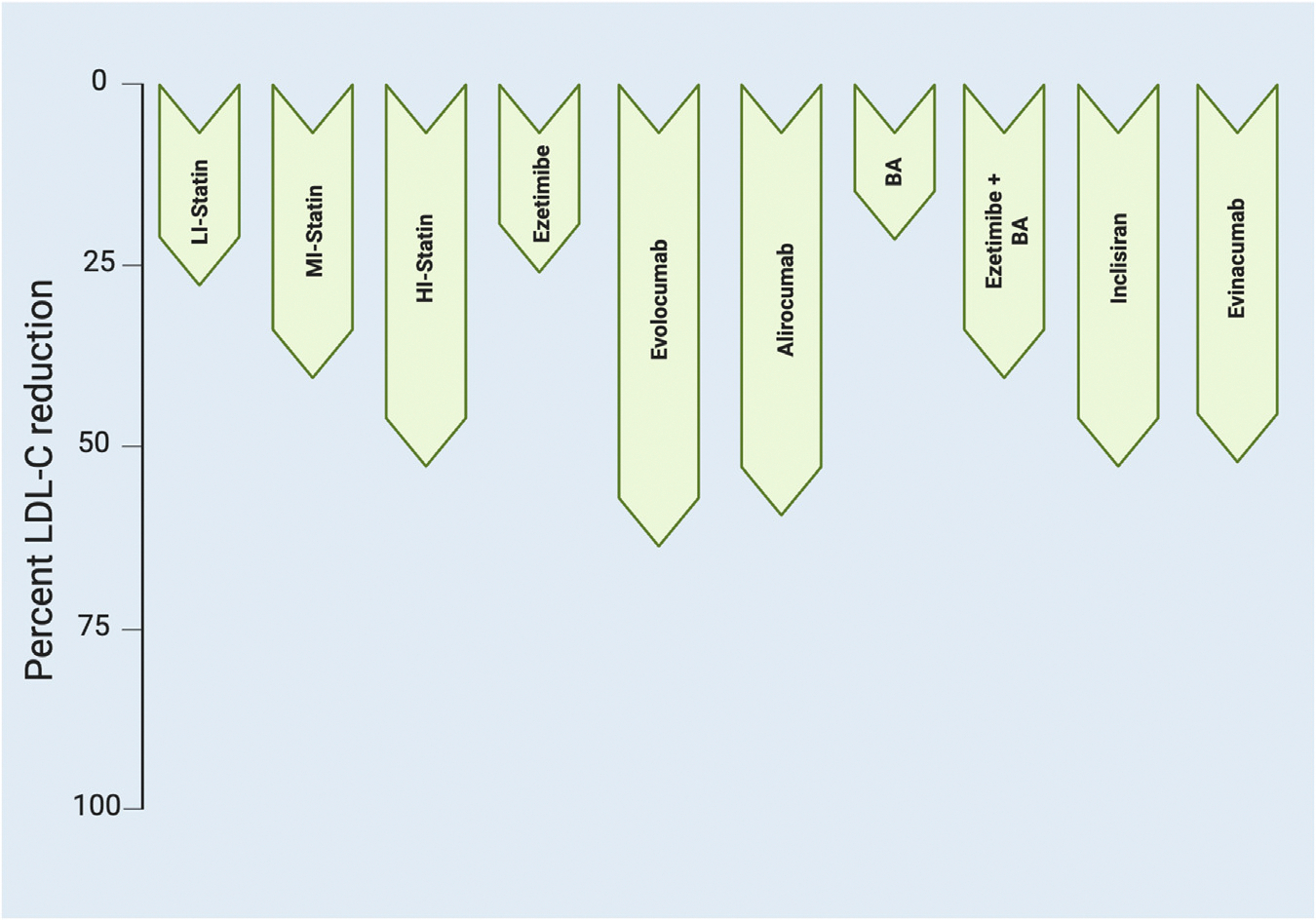
Mean percent reduction in LDL Cholesterol in response to different lipid-lowering agents. Values for non-statin therapies are based on incremental effect when added to background statin therapy according to 2022 ACC expert consensus. LI-Statin=Low-Intensity Statin, MI-Statin=Moderate-Intensity Statin, HI-Statin= High-Intensity Statin, BA=Bempedoic Acid

How does one reach very low cholesterol levels? Diet by itself can lower cholesterol but the vast majority of serum cholesterol is dictated by endogenous synthesis in the liver [24]. For this reason, pharmacologic agents are often necessary for meaningful cholesterol reduction sufficient to significantly impact cardiovascular outcomes. For three decades, statins have been the predominant choice for aggressive lipid-lowering. Research advances have provided a multitude of new drugs, including over four new molecular entities for lipid reduction (Fig. 2) [19]. Most patients do not reach very low cholesterol level on a single agent and often multiple medications are required. We review below approaches that can be used to lower LDL-C cholesterol.

### Statins

The introduction of statins marked a turning point in cholesterol management. What started with Mevastatin, first discovered from the fungus *Penicillium citrinum*, has evolved into an entire class of drugs that constitute the foundation of cardiovascular risk reduction. Statins are potent inhibitors of 3–hydroxy-3-methyl- glutaryl-coenzyme A (HMG-CoA) reductase which is the rate-limiting enzyme in the biosynthesis of cholesterol [25]. This drop in hepatic cholesterol synthesis activates SREBP-2 pathways which ultimately upregulate LDL receptor (LDLR), increasing the uptake of LDL-C from blood [26]. Statins are widely used for treating hypercholesterolemia, achieving LDL-C reductions of 30 % with low doses and up to 55 % with higher doses [27]. This dose-dependent effect makes statins a convenient option for maintaining optimal blood lipid levels without increasing side effects. High-potency statins, specifically atorvastatin (40–80 mg) and rosuvastatin (20–40 mg), have the most robust evidence base for both primary and secondary prevention of ASCVD in men and women, consistently achieving LDL-C reductions of ≥50 % and relative major adverse cardiovascular event (MACE) risk reductions of approximately 20–30 % [27].

The Heart Protection Study (HPS) demonstrated that simvastatin significantly reduced the risk of heart attack, stroke, and revascularization in high-risk individuals, even in those with relatively low baseline LDL-C levels [28]. Statins not only lower LDL-C but also exert a notable pleiotropic and anti-inflammatory effect. This is supported by observed reductions in inflammatory markers, such as C-reactive protein (CRP), with statin treatment [29]. Notably, the JUPITER trial showed that rosuvastatin reduced cardiovascular events and CRP levels in apparently healthy patients with elevated CRP and LDL-C levels under 130 mg/dL [30]. Other lines of evidence suggest that statins can exert favorable effects on a variety of cell types found in lesions, including vascular endothelial cells and immune cells, which help stabilize atherosclerotic plaques [31].

The most common side effects of statins is subjective myalgias, which occur in about 7–29 % of individuals depending on line of evidence [32], along with a modest increase in the risk of new-onset diabetes mellitus in some studies [33]. Although statins are associated with some known side effects, it is crucial to emphasize that serious adverse effects are exceedingly rare. In populations for whom statin therapy is recommended, the benefit is thought to surpass the potential risks, making them a highly effective first-line treatment option [34].

### Ezetimibe

Ezetimibe is the most widely used non-statin lipid-lowering medication [19,27] and is typically used as an add-on to statin therapy. It works by reducing cholesterol absorption in the intestines through the inhibition of the Niemann-Pick C1 Like 1 (NPC1L1) protein on the brush border of enterocytes [35]. Ezetimibe has been shown to lower LDL-C levels by up to 20 %, with a low incidence of side effects, making it a suitable option for combination therapy when target LDL-C levels have not been achieved with high-dose statins [36]. The IMPROVE-IT trial demonstrated that adding ezetimibe to statin therapy significantly reduced cardiovascular events compared to statins alone, particularly in high-risk patients, such as those with a history of acute coronary syndrome [37]. The addition of ezetimibe to simvastatin resulted in a modest 6 % relative reduction in MACE (absolute risk reduction 2 %, NNT ≈ 50 over 7 years) compared to simvastatin alone [36], underscoring that high-dose, high-potency statin therapy should generally precede ezetimibe in the treatment algorithm. Ezetimibe is especially useful for patients who are statin-intolerant or require more intensive LDL-C reduction but cannot tolerate high-dose statins. Its favorable side-effect profile, coupled with its efficacy in further lowering LDL-C, makes ezetimibe a valuable addition to the cholesterol-lowering armamentarium.

### PCSK9 inhibitors

Proprotein convertase subtilisin/kexin type 9 (PCSK9) inhibitors have an established role as adjunct therapies for cardiovascular risk reduction. Their development was inspired by evidence from genetic studies associating the PCSK9 gene locus to a novel variant of familial hypercholesterolemia [38]. Similarly, sequence variation studies showed lower risk of Coronary Heart Disease (CHD) in PCSK9 variants with low LDL-C levels [39]. The first FDA approved iteration of these drug are subcutaneously injected monoclonal antibodies–alirocumab and evolocumab. They exert their function by inhibiting the PCSK9 protein which normally binds LDLR, promoting its degradation in lysosomes. Thus, PCSK9 inhibitors facilitate hepatic LDL-C uptake by increasing LDLR density on the cell surface [40]. Adding alirocumab on top of optimal therapy showed a 62 % reduction in LDL-C throughout the ODYSSEY trial [41], while evolocumab yielded a 59 % reduction in the FOURIER trial [42]. Both FOURIER and ODYSSEY Outcomes were conducted exclusively on backgrounds of maximal-dose atorvastatin or rosuvastatin [41,42], reinforcing that PCSK9 inhibitors are best viewed as adjuncts after high-potency statin optimization rather than first-line monotherapy. Despite their efficacy in reducing cardiovascular events, the high cost of PCSK9 inhibitors has been a significant barrier to widespread use. Cost-effectiveness analyses of these injectable therapies suggest that they are economically justified for certain populations [43].

Inclisiran, a small interfering RNA (siRNA) therapy administered subcutaneously every six months, leverages the RNA-induced silencing complex (RISC) to degrade PCSK9 mRNA, resulting in approximately 48–50 % LDL-C reductions as evidenced in ORION-10 and ORION-11 trials [44,45]. Oral PCSK9 inhibitors, including MK-0616, represent a new class of lipid-lowering agents, providing significant LDL-C reductions (41–61 %) with favorable tolerability, as demonstrated in early-phase trials, with ongoing phase 3 trials evaluating their long-term efficacy and safety [46].

### Bempedoic acid

Another drug acting in the same biochemical pathway as statins is bempedoic acid. By targeting ATP-citrate lyase—an enzyme upstream of HMG-CoA reductase— this novel agent interrupts de novo cholesterol synthesis in hepatocytes. Unlike statins, bempedoic acid is a prodrug that requires activation by very-long-chain acyl-CoA synthetase 1, an enzyme primarily found in hepatocytes [47]. This liver-specific activation can mitigate the muscle-related side effects seen with statins, making bempedoic acid particularly beneficial for patients who are statin-intolerant. In the CLEAR trial, it demonstrated approximately a 21 % reduction in LDL-C levels and 13 % reduction in MACE [48].

### ANGPTL3 inhibitors

Another newly discovered class of lipid-lowering drugs targets angiopoietin-like protein 3 (ANGPTL3), a key regulator of lipoprotein metabolism. By inhibiting ANGPTL3, these therapies boost lipoprotein lipase activity, leading to reductions in LDL-C, triglycerides, and even high-density lipoprotein (HDL) cholesterol levels [49]. Evinacumab, a monoclonal antibody against ANGPTL3, has shown promising results in patients with severe or refractory dyslipidemias, including those with familial hypercholesterolemia. In clinical trials, patients treated with the maximum dose of evinacumab experienced more than a 50 % reduction in LDL-C, highlighting its potential for individuals who fail to achieve adequate LDL-C lowering with current standard-of-care treatments. Although longer-term data are still needed, ANGPTL3 inhibitors could prove particularly valuable for these high-risk patient populations [50].

## Potential benefits of very low LDL-C

### Insights from genetic studies

Naturally low LDL-C cholesterol levels are observed at certain life stages and in specific genetic conditions, often without evident harm to cardiovascular or developmental health. For instance, very low LDL-C levels naturally occur in early infancy, with LDL-C averaging around 31 mg/dL at birth, gradually rising to approximately 65 mg/dL by 2 months and reaching about 83 mg/dL by 15 months [51]. These low levels are physiologically normal and do not negatively impact health.

Furthermore, data from genetic lipid disorders reinforce the cardiovascular benefits of low LDL-C: for example, individuals with Familial Hypobetalipoproteinemia (FHBL) and abetalipoproteinemia (both associated with APOB mutations) often exhibit LDL-C levels as low as 20 mg/dL and markedly lower rates of coronary heart disease [52–54]. Conversely, in Familial Combined Hypolipidemia (FCH), which is linked to loss-of-function mutations in the ANGPTL3 gene, similarly low LDL-C levels are observed without the adverse hepatic sequelae—such as hepatic steatosis, cirrhosis, or elevated transaminases—that can occur in FHBL [55,56]. These genetic examples underscore the nuanced relationship between very low LDL-C levels and clinical outcomes, highlighting the importance of context-specific evaluations when considering aggressive LDL-C reduction.

Mendelian randomization analyses further underscore these findings by demonstrating that lifelong genetically-determined low LDL-C, including but not limited to PCSK9 loss-of-function mutations (e.g., PCSK9^142X^ or PCSK9^679X^ nonsense mutations), is consistently associated with significantly reduced coronary heart disease incidence [39,57,58]. For instance, PCSK9 mutations alone can lower mean LDL-C by approximately 28 %, correlating with up to an 88 % reduction in coronary heart disease risk [39]. However, a broad array of other genetic variants similarly reinforces the causal relationship between lifelong low LDL-C and reduced cardiovascular risk. Collectively, these genetic findings reinforce the concept that sustained low LDL-C levels confer substantial and long-term cardiovascular protection.

### Clinical trial evidence supporting benefits of LDL-C goals

The clinical benefits of aggressive LDL-C lowering have been demonstrated through several landmark clinical trials. The IMPROVE-IT trial provided initial evidence of incremental benefit from adding ezetimibe to statin therapy [59]. Similarly, the ODYSSEY Outcomes trial demonstrated substantial benefits from further LDL-C lowering through the addition of alirocumab to maximally tolerated statin therapy in 18,924 patients post-acute coronary syndrome. The alirocumab group achieved LDL-C levels as low as 38–53 mg/dL, significantly reducing cardiovascular events and mortality compared to placebo, a major adverse cardiovascular events (MACE) risk reduction from 14.2 % to 12.0 % over a maximum of 5 years follow up [60].

The FOURIER trial further validated these findings by examining evolocumab in 27,564 high-risk patients. Achieving extremely low median LDL-C levels of 30 mg/dL, evolocumab significantly reduced primary cardiovascular endpoints by 15 % and key secondary endpoints by 20 % [61]. Notably, these reductions continued to increase over time, reinforcing the cumulative benefit of sustained low LDL-C management [62]. A meta-analysis by Sabatine et al. synthesized these findings and additional clinical trial data, emphasizing the broad and consistent cardiovascular benefits of LDL-C reduction. This analysis demonstrated a 21 % relative reduction in MACEs per 1 mmol/L (38.7 mg/dL) LDL-C reduction, even in patients with baseline LDL-*C* ≤ 70 mg/dL [63]. Remarkably, these benefits extended to on-treatment LDL-C levels as low as 21 mg/dL, without increased risks of serious adverse events such as diabetes, hemorrhagic stroke, or cancer [63]. Another analysis of the FOURIER trial aimed to address whether there is continued cardiovascular benefit from lowering LDL-C to levels <40 mg/dL. The data show that the cardiovascular benefits of LDL-C lowering persist regardless of how low the baseline LDL-C is, with robust risk reductions observed even when a substantial portion of the reduction happens below the 40 mg/dL threshold [64].

Interestingly, in a recent post-hoc analysis of FOURIER, patients with autoimmune/inflammatory disease (∼3 % of the cohort) displayed a ∼60 % LDL-C reduction with evolocumab (no change in hsCRP) and experienced a 42 % reduction in the primary cardiovascular endpoint versus 14 % in those without inflammation (P-interaction=0.066) [65]. Comparable benefits were seen for cardiovascular death/MI/stroke (58 % vs. 19 %; P-interaction=0.022) [65]. This suggests that patients with systemic inflammation may derive even greater benefit from intensive LDL-C reduction through PSCK9 inhibition.

Pharmacological LDL-C lowering therapies have demonstrated compelling benefits in both regressing coronary plaque and enhancing plaque stability through favorable changes in plaque composition, including increased fibrous cap thickness. For instance, high-intensity statin therapy, as shown in the REVERSAL trial [66], significantly reduced atheroma volume and improved plaque morphology. In the STABLE study, both high-intensity (rosuvastatin 40 mg) and moderate-intensity (rosuvastatin 10 mg) regimens resulted in favorable plaque modifications, with decreases in necrotic core volume, overall plaque volume, and the rate of thin-cap fibroatheroma [67]. Moreover, imaging data from the GLAGOV trial [68] demonstrated that aggressive LDL-C reduction (achieving a mean LDL-C of 36.6 mg/dL versus 93 mg/dL with standard therapy) leads to significant plaque regression using PCSK9 inhibitors on a background of statin-therapy. Similarly, this further enhances plaque remodeling and fibrous cap stabilization as highlighted by the PACMAN-AMI trial [69], underscoring the critical importance of achieving very low LDL-C levels to mitigate cardiovascular risk.

Together, findings from clinical trials and observational imaging studies lend support to the notion that more aggressive lipid-lowering strategies at least till an LDL-C level of <40 mg/dL and perhaps up to 20 mg/dl can be of value in mitigating cardiovascular risk [21].

## Potential risks of very low LDL-C

### Stroke & intracranial hemorrhage

A number of epidemiological studies highlighted the potential for increased risk of intracranial hemorrhage (ICH) with low LDL-C [70–72]. Interestingly, this relationship has been shown in a number of Mendelian Randomization studies as well [73,74]. However, in patients without a prior history of cerebrovascular disease, the risk of hemorrhagic stroke associated with statin therapy is small and consistently nonsignificant [75]. In addition, larger well-controlled randomized trials dispute the concern of hemorrhagic stroke with low LDL-C levels [76,77]. For example, SPARCL (Stroke Prevention by Aggressive Reduction of Cholesterol Levels) [77] and TST (Treat Stroke to Target) [76], found no significant relationship between aggressive LDL-C reduction and increased hemorrhagic stroke risk. Specifically, these trials indicated that poorly controlled hypertension and a history of previous hemorrhagic stroke, rather than LDL-C levels or statin therapy itself, correlated significantly with increased risk of hemorrhagic stroke [76,77]. Furthermore, studies evaluating PCSK9 inhibitors such as evolocumab and alirocumab have also demonstrated no significant increase in hemorrhagic stroke risk, even with aggressive LDL-C reduction to levels below 20 mg/dL. For instance, the FOURIER trial reported a hazard ratio of 1.16 (95 % CI, 0.68–1.98) [78] and ODYSSEY OUTCOMES trial a hazard ratio of 0.83 (95 % CI, 0.42–1.65) [79], further supporting the safety of aggressive LDL-C lowering strategies. It is important to note that both ODYSSEY and FOURIER trials excluded subjects with prior history of hemorrhagic stroke. The strong correlation between lower LDL-C and reduced overall stroke risk—primarily due to decreased ischemic stroke incidence—is thought to outweigh any potential concerns about hemorrhagic stroke. In a recent large scale meta-analysis on hemorrhagic stroke risk, the estimated number needed to harm with statins was 3333 in an average span of 6.7 years [80]. This is in comparison to number needed to treat of 49 to prevent one ischemic event within the span of 5 years [81]. Notably, a registry of 502 ICH survivors found LDL-*C* < 70 mg/dL doubled recurrent ICH risk (AHR 1.99; 95 % CI 1.06–3.73), predominantly in those with cerebral amyloid angiopathy or intrinsically low LDL-C, while statin use itself carried no excess risk (AHR 1.07; 95 % CI 0.57–2.00) [82], underscoring the importance of individualized LDL-C management in patients with prior ICH. Although further research is needed to illuminate the precise risks of very low LDL-C in patients with a history of ICH, the overwhelming majority of evidence does not support a concern of increased risk for the majority of patients.

### Cognitive function and dementia

Observational studies and small randomized clinical trials raised the question of whether very low LDL-C could impact cognitive function [83,84]. However, long-term data from the EBBINGHAUS study, which evaluated cognitive outcomes in patients treated with PCSK9 inhibitors and statins (median LDL-C of 35 mg/dL), found no association between LDL-C lowering and cognitive impairment over a follow-up period of up to seven years [85,86]. Strengths of this study includes intensive testing of cognitive function. Data from the ODYSSEY trial also reinforces the absence of any relationship between cognitive dysfunction and LDL-C levels lower than 25mg/dL in response to the PCSK9 inhibitor alirocumab [87]. Additionally, the 2023 American Heart Association statement reviewing observational and randomized trial data concluded that there is no significant relationship between LDL-C reduction and cognitive dysfunction. These findings reinforce the safety of aggressive LDL-C lowering therapies in preserving cognitive function [88].

### Vitamin E and steroid hormones

Since the LDL-C particle serves as a carrier of fat-soluble vitamins and cholesterol-derived steroid hormones, there is a theoretical risk that very low LDL-C levels could affect vitamin E status and steroidogenesis. The DESCARTES study examined vitamin E metabolism in patients treated with evolocumab and found that while absolute serum and LDL-C vitamin E levels decreased, cholesterol-normalized vitamin E levels remained stable. This suggests that the observed reductions in vitamin E are merely reflective of the lower lipid content of LDL-C particles, rather than an actual vitamin E deficiency. Furthermore, adrenal function studies in patients treated with rosuvastatin and evolocumab found no impairment in cortisol production, evidence of adrenal insufficiency or sex hormone production, even in those achieving extremely low LDL-C levels (<15 mg/dL) [89]. Similarly, previous studies on gonadal steroidogenesis in both male and female patients have not demonstrated any clinically significant changes in testosterone, gonadotropins, or ovarian function [90,91], reinforcing the lack of an adverse impact on endocrine health with LDL-C lowering therapy.

### Cataracts

It has been hypothesized that very low LDL-C levels could increase the risk of cataracts, given that lens fibers have a high cholesterol content that is essential for maintaining transparency. While some small animal and human studies including the HOPE-3 trial have suggested a possible link between low cholesterol and cataract development [92], data from statin and PCSK9 inhibitor trials found no increase in cataract risk with either class of therapy. However, patients on alirocumab who had LDL-C <25 mg/dl had a slightly increased risk which has been theoretically attributed to possible acceleration of underlying aging and metabolic syndrome [93]. Additionally, there was no observed association between achieved LDL-C levels and cataract incidence, suggesting that concerns regarding cataract formation should not deter the pursuit of aggressive LDL-C lowering [94].

## A suggested approach to navigating very low LDL-C in clinical practice

The overall balance of evidence suggests that achieving very low LDL-C can be beneficial despite potential risks. As a general rule, we agree with target goals outlined in AHA/ACC and ESC guidelines and strive to achieve these goals. Observational data suggest that most patients remain on their initial statin dose [95]. Accordingly, it may be more effective to initiate treatment at a higher dose and, if intolerance arises, titrate downward— rather than up-titrating from a low starting dose. In high-risk patients with multiple comorbidities and LDL-C approaching 50 mg/dL, the marginal gains from adding another daily lipid-lowering pill must be balanced against the risk of diminished overall adherence. Studies have shown that complex regimens can lead to medication non-adherence [96] —one-half of patients do not adhere to cardiovascular prevention treatment [97]. Clinicians should prioritize therapies with proven incremental benefit (e.g. statins), and use fixed-dose combination pills when available to minimize polypharmacy [96]. Regular medication reconciliation, patient education, and a shared-decision making approach can lead to more favorable patient outcomes [96].

From our experience, patients on multiple therapies can sometimes drop their LDL-C to the very low LDL-C range (20–40 mg/dl). In this context, we do not proactively reduce or hold lipid-lowering therapies. We have an informed discussion on potential benefits and risks in a collaborative approach with patients. In addition, in select groups of patients at very high risk for future events (e.g. recurrent events despite achieving lipid goals), we aim for very low LDL-C that approaches 20 mg/dL. While it is conceivable the benefits in this range could be incremental or of unclear significance, other lines of evidence suggests that lowering LDL-C in this range could be clinically meaningful [64]. Finally, from our experience younger patients at high risk (e.g., Familial hypercholesterolemia with clinical event) and those with recurrent hemorrhagic bleeds may be particularly concerned about unknown or long-term risks of very low LDL-C. We don’t believe that such scenarios epitomize an absolute contraindication to achieving very low LDL-C. In partnership with patients, we individualize the potential of benefits and risks of very low LDL-C based on a shared-decision making approach as well as understanding of patient needs, values and preferences.

## Conclusion and future direction

Overall, empirical evidence shows that intensive LDL-C lowering delivers significant cardiovascular protection with minimal safety concerns. The magnitude of clinical benefit achieved through aggressive lipid reduction outweighs the small, at times theoretical hazards associated with very low LDL-C. Therefore, on balance, the evidence supports a more aggressive approach to LDL-C reduction for mitigating cardiovascular risk. Statins remain the cornerstone of therapy, while newer agents—including PCSK9 inhibitors, ezetimibe, bempedoic acid, and ANGPTL3 inhibitors—have expanded our options for patients who are either statin-intolerant or require additional lipid-lowering to achieve optimal targets. Genetic studies and long-term clinical trials demonstrate that very low LDL-C levels not only confer enhanced protection against atherosclerotic events but do so without compromising endocrine function, cognitive health, or increasing hemorrhagic stroke risk in most patient populations. Analyses of routine clinical care records indicate that fewer than one in four ASCVD patients achieved the current guideline goal of LDL–C < 55 mg/dL [98], despite widespread statin use and evidence that adding non–statin therapies improves goal attainment. This treatment gap underscores the need to intensify therapy—routinely using high–intensity statins and incorporating combination regimens—to help more patients reach recommended LDL–C targets. For example, early in-hospital initiation of PCSK9-inhibitors cut median LDL-C from 137 to 43 mg/dL and halved MACE over 11 months [99], underscoring the “strike early, strike strong” approach. Furthermore, emerging genetic insights—such as the development of polygenic risk score (PRS) — offer the potential to tailor LDL-C targets based on an individual’s genetic profile, particularly for those with heightened cardiovascular sensitivity to lipid levels. By utilizing these PRS, clinicians can determine whether a more intensive LDL-C lowering strategy is necessary for patients with a heightened genetic risk of coronary artery disease, thereby fine-tuning treatment to effectively manage risks associated with endothelial dysfunction [100]. While further research is needed in specific scenarios—such as patients with prior intracerebral hemorrhage—these findings collectively reinforce the safety and efficacy of aggressive LDL-C lowering strategies. As novel therapies continue to emerge, clinicians can tailor treatment to individual risk profiles, balancing the proven benefits of early and intensive LDL-C reduction against any residual uncertainties. This personalized, evidence-driven approach stands to significantly reduce the global burden of cardiovascular disease and improve long-term patient outcomes.
